# Precision Health for Chagas Disease: Integrating Parasite and Host Factors to Predict Outcome of Infection and Response to Therapy

**DOI:** 10.3389/fcimb.2020.00210

**Published:** 2020-05-08

**Authors:** Santiago J. Martinez, Patricia S. Romano, David M. Engman

**Affiliations:** ^1^Laboratorio de Biología de Trypanosoma cruzi y la célula hospedadora—Instituto de Histología y Embriología “Dr. Mario H. Burgos,” (IHEM-CONICET- Universidad Nacional de Cuyo), Mendoza, Argentina; ^2^Department of Pathology and Laboratory Medicine, Cedars Sinai Medical Center, Los Angeles, CA, United States; ^3^Department of Pathology and Laboratory Medicine, University of California, Los Angeles, Los Angeles, CA, United States; ^4^Departments of Pathology and Microbiology-Immunology, Northwestern University, Chicago, IL, United States

**Keywords:** chagas disease, *Trypanosoma cruzi*, therapy, outcome of infection, precision health

## Abstract

Chagas disease, caused by the infection with the protozoan parasite *Trypanosoma cruzi*, is clinically manifested in approximately one-third of infected people by inflammatory heart disease (cardiomyopathy) and, to a minor degree, gastrointestinal tract disorders (megaesophagus or megacolon). Chagas disease is a zoonosis transmitted among animals and people through the contact with triatomine bugs, which are found in much of the western hemisphere, including most countries of North, Central and South America, between parallels 45° north (Minneapolis, USA) and south (Chubut Province, Argentina). Despite much research on drug discovery for *T. cruzi*, there remain only two related agents in widespread use. Likewise, treatment is not always indicated due to the serious side effects of these drugs. On the other hand, the epidemiology and pathogenesis of Chagas disease are both highly complex, and much is known about both. However, it is still impossible to predict what will happen in an individual person infected with *T. cruzi*, because of the highly variability of parasite virulence and human susceptibility to infection, with no definitive molecular predictors of outcome from either side of the host-parasite equation. In this Minireview we briefly discuss the current state of *T. cruzi* infection and prognosis and look forward to the day when it will be possible to employ precision health to predict disease outcome and determine whether and when treatment of infection may be necessary.

## *Trypanosoma cruzi* and Chagas Disease

Chagas disease, American trypanosomiasis, is caused by infection with the protozoan parasite *Trypanosoma cruzi* which displays a complex life cycle involving human and animal hosts as reservoirs of disease and triatomine insects of the Reduviidae family as vectors. Although the route of infection was originally felt to be restricted to contamination of the wound or mucous membrane with *T. cruzi*-contaminated excreta of hematophagous insects, other forms of transmission are also important, including oral infection through consumption of food and drink contaminated with the parasite, blood transfusion, organ transplantation, and congenital infection (Moncayo, 2003; Coura, 2014; Dolhun and Antes, 2016; Alarcón de Noya et al., 2017). Although 6–7 million infected individuals live in the Americas (WHO, 2020), migration of *T. cruzi*-infected people throughout the world, many of whom are unaware of being infected, has contributed to the globalization of the disease (Steverding, 2014). Of the 238,000 infected people which are believed to reside in the United States, mostly immigrants from South America (Meymandi et al., 2017), 30,000 are found in Los Angeles, where Dr. Sheba Meymandi oversees a large Chagas clinic and a Center of Excellence for Chagas Disease (Meymandi, 2020). A few dozen cases of vector-borne transmission have been documented in the United States, although infection is widespread in wild animals throughout the southern half of the country (Montgomery et al., 2016; Kruse et al., 2019). The lack of an effective vaccine against *T. cruzi*, and the moderate effectiveness and toxicity of first-line drugs aggravate the situation (Schaub et al., 2011; Nunes et al., 2013; Rodríguez-Morales et al., 2015). Considering these aspects of epidemiology, continued surveillance of insects and wild animals, continued screening of the blood supply, and perhaps implementing screening of women of childbearing age will help to reduce transmission of *T. cruzi* through various routes.

In the human host, *T. cruzi* trypomastigotes, the infective forms of the parasite, can enter a wide variety of host cells. Trypomastigotes then differentiate into amastigotes which replicate in the cytoplasm and differentiate back to trypomastigotes again, which lyse the host cell membrane and exit the cell to continue the infectious cycle in the human. Cardiac and smooth muscle tissues are preferential cellular targets of *T. cruzi*. The adverse sequelae of infection described below depend on the tissues and organs involved, which is a highly variable and unpredictable factor. Chagas disease is highly complex. While traditionally considered as having acute, indeterminate (chronic–asymptomatic) and chronic (symptomatic) phases, this illness is highly heterogeneous and best considered to be a unique illness for each patient (Bonney et al., 2019). Most infected individuals live normal lives and eventually die of causes other than Chagas disease, completely unaware of their lifelong infection, whereas around 30% of infected people develops clinical manifestations. The acute phase of *T. cruzi* infection, lasting 4–8 weeks, often has no associated symptoms, despite the fact that the parasite is replicating and spreading throughout the body (Bastos et al., 2010; De Bona et al., 2018). In the case of vector transmission, it is possible to see Romaña's sign around 5% of the time, when parasites deposited by the triatomine on the face enter the conjunctiva, leading to periorbital inflammation and edema. Chagoma, an inflammatory skin lesion at the site of the insect bite, is also occasionally observed (Bastos et al., 2010). In most cases, however, acute infection is not recognized due to the non-specificity of signs and symptoms (fever, anorexia, and/or flu-like symptoms like body ache). In very rare cases acute infection leads to sudden death, due to parasitization of the cardiac conduction system and a fatal dysrhythmia. In most people, parasite-specific adaptive immunity develops, keeping overall tissue parasitosis and blood parasitemia at very low levels for life. In contrast, approximately one-third of infected individuals develop cardiomyopathy or, to a lesser degree, mega disease of the esophagus or colon, occurring many years after infection. Disease pathogenesis is extremely complex with multiple known and proposed mechanisms of tissue-specific damage. Current data highlight the persistence of parasites in cardiac tissue as a key factor to disease progression, whether by anti-parasite immunity, autoimmunity or other mechanisms, suggesting that reduction of parasitosis through trypanocidal treatment is key to combatting the illness (Hyland et al., 2007; Viotti et al., 2009; Bastos et al., 2010; Bocchi et al., 2017; Bonney et al., 2019). We have recently reviewed pathogenesis (Bonney et al., 2019) and will not discuss this further in this review.

## Treatment of *Trypanosoma cruzi* Infection

### Current Treatment for Chagas Disease

*Trypanosoma cruzi* infection is treated with Benznidazole (BNZ) or Nifurtimox (NFX), nitroimidazole compounds that have been used for decades. The approach currently practiced by most is to treat all acutely infected individuals, newborns with congenital infection, and anyone under 50 years of age. Further, all immunocompromised individuals such as those with HIV/AIDS or other immunosuppressive disorders or treatments, should be treated to prevent reactivation of chronic infection, normally maintained at very low levels by effective adaptive immunity (Pinazo et al., 2013). BNZ is administered to adults a dose of 5–8 mg/kg/day for 60 days. Children's doses are somewhat higher because they are more tolerant to the drugs and show quicker resolution of the common hepatic and renal toxicity upon drug cessation. Adults over 50 years of age with chronic *T. cruzi* infection should be considered individually, balancing the potential benefits and risks based. BNZ treatment is contraindicated for pregnant women and people with significant hepatic and renal illness (WHO, 2020). NFX is recommended as a second line drug, only in the cases of BNZ failure and in the absence of neurological and psychiatric disorders. NFX is administered at 8–10 mg/kg/day for 90 days in adults, and at 15–20 mg/kg/day for 90 days in children (Bern et al., 2007).

Although there are cases in which BNZ has been found to be more effective than NFX, both in the laboratory and in patients, the reasons for these differences are not known (Olivera et al., 2017; Crespillo-Andújar et al., 2018). Limitations of BNZ monotherapy includes the lower probability of parasitological cure in cases of chronic infection in contrast to the high probability of parasitological cure in the acute phase when treatment is maintained for the entire 60 day treatment period (Meymandi et al., 2018). It is also possible that BNZ-resistant *T. cruzi* clones emerge after partial treatment (Hughes and Andersson, 2017). Finally, the relatively short half-life of the drug (about 12 h), the low penetration of some tissues (Perin et al., 2017) and the occasional serious side effects are additional limitations. These adverse side effects are well-known, and include allergic dermatitis, peripheral neuropathy, anorexia, weight loss, and insomnia (Castro and Diaz de Toranzo, 1988). When they do develop, these side effects occur early in treatment and often become intolerable, causing patients to abort treatment; this can occur in up to 40% of individuals (Castro and Diaz de Toranzo, 1988; Castro et al., 2006; Viotti et al., 2009).

There have been a number of attempts to improve BNZ and NFX therapy, both to increase efficacy and to reduce toxicity, by decreasing the daily dose, giving the drug intermittently, or preemptively treating potential side effects (Bastos et al., 2010; Álvarez et al., 2016; Morillo et al., 2017; Rassi et al., 2017; Cardoso et al., 2018). During the last decade, two important randomized clinical trials were conducted to evaluate the capacity of BNZ to modulate the evolution of Chagas heart disease in adult patients with established cardiomyopathy—the BENEFIT study (Morillo et al., 2015) and the TRAENA trial (Riarte, 2012). Both used a dose of 5 mg/kg/day of BNZ or placebo for 60 days and patient follow up over 5–10 years. Both found that BNZ was able to significantly reduce parasitemia and parasite-specific serum antibodies. However, these trials also showed that BNZ did not significantly reduce progression of clinical cardiac disease through 5 years of follow-up. Additional studies confirmed the low efficacy of BNZ to prevent progression of cardiomyopathy in patients with documented heart disease (Rassi and Rassi, 2010; Rassi et al., 2017). What these trials did not address is the potential benefit of therapy to indeterminate patients. Can drug treatment prevent the development of cardiomyopathy in chronically infected people with no cardiac disease? A retrospective study addressed this directly and showed that treatment with BNZ prevents the development of ECG alterations and decreases parasite-specific antibody titers in indeterminate patients (Fragata-Filho et al., 2016). Taken together, and considering additional studies (Villar et al., 2014; Pérez-Molina et al., 2015), these data suggest that trypanocidal therapy benefits acutely infected individual and chronically infected people who have not yet developed clinical heart disease.

### Approaches to Improve Treatment of Chagas Disease

Research on new treatments involves two main strategies: a search for new candidate drugs that are more effective and less toxic to replace BNZ, and a search for adjunctive agents that can either increase the efficacy of BNZ/NFX or reduce their doses to prevent adverse effects. Typically, compounds tested for efficacy as monotherapy are also tested in combination with BNZ/NFX.

The main approaches to preclinical drug discovery for *T. cruzi* drugs involve seven main groups of inhibitors: (1) inhibitors of ergosterol biosynthesis (e.g., posaconazole and other antifungal azoles), (2) trypanothione metabolism (amiodarone and dronedarona), (3) pyrophosphate metabolism (biphosphonates), (4) cruzipain inhibitors (K777 and derivates), (5) calcium metabolism (amiodarona, dronedarona), (6) protein and purine synthesis inhibitors, and (7) compounds that impair the redox metabolism (nitroaromatic compounds like BNZ, NFX and fexinidazole). Unfortunately, only a few clinical trials for treatment are ongoing or were performed recently for these candidates (Apt, 2010; Sales Junior et al., 2017).

Inhibitors of ergosterol biosynthesis affect the production of the parasite cell membrane and show trypanocidal effects, similar to what they do in fungi. For *T. cruzi*, a number of antifungals drugs have been found to have good *in vitro* and *in vivo* efficacy (Bustamante et al., 2014; Molina et al., 2014; Torrico et al., 2018), both as single agents and in combination with BNZ. Posaconazole, for example, demonstrated trypanocidal activity particularly in combination with BNZ (Bustamante et al., 2014). However, in clinical trials, no advantage was observed with the combined therapy vs. BNZ monotherapy (Morillo et al., 2017). In addition, posaconazole showed no curative effects in patients on its own (Molina et al., 2014). Ravuconazole and E-1224, a ravuconazole prodrug with better drug absorption and bioavailability, are antifungal azoles with potent *in vitro* activity against *T. cruzi*. However, E-1224 failed to show sustained efficacy 1 year after treatment in comparison with BNZ and presented some safety issues at high doses (Torrico et al., 2018).

Besides antifungals azoles, the most advanced candidates in clinical trials are amiodarone and fexinidazole, which have ongoing or completed Phase II clinical studies, respectively. One advantage of amiodarone is its potential dual role in patients with cardiomyopathy since it is an antiarrhythmic drug as well as a potent and selective anti-*T. cruzi* agent (Benaim and Paniz Mondolfi, 2012). Dronedarone, a derivate of amiodarone developed to reduce thyroid toxicity, showed a better profile at a lower dose, and will hopefully be tested in a clinical trial soon (Benaim et al., 2012).

The Drugs for Neglected Diseases initiative (DNDi) has actively chosen to investigate nitroaromatic compounds. Their investigations have proved fruitful, resulting in a trypanosomatid portfolio that contains several agents. The DNDi portfolio published in December 2019 lists fexinidazole as in a Phase IIa clinical trial, whereas new BNZ regimens are in Phase IIb/III. Fexinidazole can induce high levels of parasitological cure in mice infected with BNZ-susceptible, partially resistant and resistant *T. cruzi* strains in acute and chronic experimental Chagas models (Bahia et al., 2012). These and other data have encouraged DNDi to include fexinidazole in clinical studies. In addition, the BENDITA (Benznidazole New Doses Improved Treatment & Associations) trial showed that a BNZ 2-week treatment course for adult patients with chronic Chagas disease displayed similar efficacy and significantly fewer side effects than the standard treatment duration of 8 weeks, when compared to placebo (DNDi, 2019). DNDi will now continue to work with national programs, partners, and health ministries of endemic countries to confirm these results and encourage the necessary steps to register the new regimen.

Other interesting strategies in preclinical studies are nanoparticle therapy and natural compounds. Considering that a major disadvantage of BNZ is its high toxicity, recent work has employed nanotechnology to attempt deliver this drug in an effective but safe way. The development of nanoparticles for drug delivery is an area of great promise. The earliest particles investigated were liposomal formulations of BNZ, which were developed to target the drug to the liver (Morilla et al., 2004). Since that time a variety of particles have been tested, including polymethacrylate interpolyelectrolyte complexes (García et al., 2018) and the amphipathic poloxamer P188 (Scalise et al., 2016). While these formulations were tested in different *in vitro* and *in vivo* systems, they show great promise in delivering BNZ and other trypanocidal agents to parasites and parasitized cells at lower effective BNZ doses with lower associated toxicity.

Natural compounds constitute a newer but nonetheless active area of Chagas drug discovery. Many plants extract display trypanocidal properties, with some demonstrating activity more potent than BNZ or NFX. Like other drugs, natural trypanocides can be useful either as independent agents, or through enhancing the activities of BNZ or NFX by enhancing their uptake by host cells, killing of intracellular amastigotes, or reducing toxicity. Drug repurposing is also being applied to *T. cruzi* as it is to many infectious and non-infectious diseases (Bellera et al., 2015). Some effective drugs can come from unlikely places, like agents used in cancer chemotherapy (Epting et al., 2017), antivirals, antibiotics, and cardiac medicines (Bellera et al., 2015).

Despite much research by hundreds of researchers over several decades, we still do not have an agent or regimen that is superior to BNZ/NFX for the treatment of *T. cruzi* infection. Several candidates showed good trypanocidal activity *in vitro*, but fail preclinical or clinical trials. There are many factors determining the outcome of infection and susceptibility of the parasite to treatment beyond what can be measured through typical studies. In the rest of this Mini Review we discuss other aspects of the host-pathogen interaction that impact the outcome of infection and treatment, which should be considered in whether, when and how to treat infection.

## Outcome of *T. cruzi* Infection and Efficacy of Treatment Depend on Many Factors

It is difficult to extrapolate the results of *in vitro* tests to *in vivo* animal studies and even harder to extend those results to humans. Besides being human, people are highly heterogeneous genetically, and physiologically and respond to most challenges and interventions, including infections and drug treatments, with great variation; this can lead to treatment failures (Francisco et al., 2015). Although success of any treatment can be measured by the reduction of parasitemia and even of parasite-specific serum antibodies, success is ultimately measured by reduction in the development of long-term sequelae such as cardiomyopathy and megacolon. As mentioned above, treatment with intermittent low doses of BNZ in patients with established chagasic cardiomyopathy significantly reduced parasitemia, but not progression of cardiomyopathy (Morillo et al., 2015). Also *T. cruzi* displays a high degree of genetic and pathogenetic heterogeneity and are commonly present as mixtures of distinct parasite clones in a single infected triatomine or infected host (Pronovost et al., 2018). However, it is theoretically possible to predict the outcome of infection—subclinical for life, cardiomyopathy, mega disease—if we knew more about the genetic and physiologic basis of parasite virulence (broadly defined) and host susceptibility (also broadly defined). We are a long way from this understanding today. Variability in host physiologic factors such as nutrition, immune status, existence of coinfections, etc., further complicate the issue. The balance among host genetics, host physiology and parasite genetics determine outcome of infection and response to treatment. A number of these are discussed below. The reader should keep in mind that these factors are ultimately based in large part on the genetics of host and parasite, which makes a systems approach to Chagas disease management possible in the future.

### Epidemiology

In the absence of other information, epidemiologic data can be of modest help in predicting the outcome of *T. cruzi* infection. Information about patient origin, possible form of transmission (insect, congenital, oral), presence of other conditions such as immunosuppressive states such as cancer, HIV coinfection, or treatment with immunosuppressive drugs, may inform patient management. Clearly, infected individuals who are immunocompromised need treatment. Other aspects of the infection, such as the location where infection takes place, and by extension the characteristics of the human and parasite populations, can be useful. An estimated two-thirds of infected Brazilians are infected with the TcII strain of *T. cruzi* (Brenière et al., 2016; Zingales, 2018), one of seven discrete typing units (DTU) TcI-TcVI, plus TcBat (Zingales et al., 2009; Lima et al., 2015). Some of these DTUs can be identified serologically (Bhattacharyya et al., 2019). TcII strains, represented by the common laboratory strain Y, generally exhibit high virulence and may produce mega disease as well as cardiac disease in chronic infection (De Oliveira et al., 2008; Oliveira et al., 2017). In contrast, people from Argentina and Bolivia frequently are infected with TcV strains and frequently develop cardiomyopathy (Zuñiga et al., 1997; Messenger et al., 2015; Quebrada Palacio et al., 2018; Zingales, 2018). Other DTUs such as TcI (e.g., Colombian) or TcVI (e.g., Tulahuen) have a tendency not to cause clinical disease and are often used in chronic indeterminate mouse models of infection (Chandra et al., 2002; Santana et al., 2014). Unfortunately, the DTU system alone is not sufficient to predict disease outcome or response to therapy since there is no single outcome associated with any given DTU. No physician would withhold drug treatment in an acutely infected individual simply based on the fact that they may be infected with one particular *T. cruzi* strain or another.

### Parasite Virulence

Virulence is a complex term in its own right. It is important to carefully define at each use. Virulence could be the capacity of *T. cruzi* to invade host cells, replicate, and emerge after host cell lysis. This leads to high parasitemia in experimental animals. It could refer to tissue tropism, with some tissue infections being more harmful to the host than others. Virulence might refer to the ability of the parasite to kill its host. At some level, considering the parasite alone, virulence is based on genetic elements. Virulence may be conferred by specific parasite surface proteins or secreted proteins that signal host cells, facilitating parasite entry and replication. Molecules from trans-sialidase and cruzipain families are well-established virulence factors of *T. cruzi* and validated targets for drug discovery. Cruzipain also participates in the modulation of the host cell immunity, highlighting the key role of the host response in the establishment and outcome of *T. cruzi* infection (Guiñazú et al., 2004; San Francisco et al., 2017). High virulence is usually defined as the ability to cause high parasitemia and/or tissue parasitosis and/or death of experimental animals (Sales-Campos et al., 2015). This is based in part on the ability to invade and/or replicate in host cells more rapidly than do low virulence isolates. Low virulence strains are more likely to cause low-level chronic infection that may never cause clinical disease (Cardillo et al., 2015). As mentioned above, the commonly used high-virulence Y strain of *T. cruzi* causes death in young C57BL/6 mice between 14 and 21 days post-infection in conjunction with maximal parasitemia (Casassa et al., 2019). By contrast, the K98 strain causes chronic infection. It should be emphasized that virulence is equally influenced by the host (Ferreira et al., 2018), as discussed in more detail below.

### Tissue Tropism

Another characteristic of *T. cruzi* is differential tissue tropism. Some isolates of this parasite have a propensity to infect certain tissues over others. This may be due to specific affinity for certain host cell surface molecules, preferential ability to replicate in some cells better than others, or specific attraction to an organ-specific vascular bed. If a person becomes infected with a myotropic strain, it is more likely that cardiac pathology or skeletal myositis will develop. On the contrary, a pantropic strain may affect many organs and promote development of megaesophagus or megacolon. Tropism can be ascertained by *in vitro* testing using different cell lines. A strain with cellular myotropism will prefer H9C2 cardiac myoblasts or human skeletal myoblasts (Jorge et al., 1986; Mirkin et al., 1997; Aridgides et al., 2013), while a pantropic strain will not have a preference, for example affecting kidney cells and embryonic fibroblasts equally (Piras et al., 1982; Jorge et al., 1986; Medina et al., 2018). Information about the tropism of parasite clone or clones could potentially be important in guiding treatment decisions, including a decision not to treat. The challenge of course is to determine potential tropism or other characteristics of a clone without having isolated and cultured parasites for laboratory study. There may be ways to do this in the future using a combination of advanced imaging and molecular approaches (see below). In the meantime, it is really not possible to predict the tissue tropism of a strain based on the region of origin or DTU.

### Drug Resistance

The differential resistance of *T. cruzi* to BNZ among isolates has been documented (Bustamante et al., 2014; Abegg et al., 2017; Vieira et al., 2018). This intrinsic resistance in some strains could explain why some patients receiving the same BNZ treatment show parasitological cure while others do not. Similar to virulence, the capacity of parasites to resist or be susceptible to a drug is genetically determined by the presence of specific factors. The *T. cruzi* Colombian and V-10 strains are highly resistant to BNZ, while the Y and Dm28c strains are partially resistant and the CL strain is highly sensitive (Filardi and Brener, 1984; Bahia et al., 2012; Reigada et al., 2019). Somewhat paradoxically, intracellular replication of some strains is enhanced by the presence of BNZ and the associated production of reactive oxygen species (Paiva et al., 2018). Several have proposed that different *T. cruzi* DTU have different resistance to BNZ and NFX (Cencig et al., 2012; Teston et al., 2013). Some DTU are more resistant to BNZ than others, although even within a single DTU there can be variability in BNZ sensitivity (Quebrada Palacio et al., 2018). Interestingly, parasite strains of different DTUs do show common BNZ susceptibility and resistance patterns (Revollo et al., 2019). Clearly there can be much greater refinement in genetic characterization of *T. cruzi* than the DTU system but it is a measure that has shown great utility in many studies. However, data from genotyping could be used to predict the susceptibility of an isolate to drug treatment. As mentioned above and discussed below, the challenge is to be able to genotype or phenotype parasites without isolating them, since in many chronically-infected patients circulating parasites are rare or absent. As a relatively crude measure of *T. cruzi* sensitivity to BNZ, quantitative PCR to detect parasite DNA in blood before and after BNZ treatment is the best we have at the moment (Britto et al., 1999; Maffey et al., 2012; Barros et al., 2017; Rodrigues-dos-Santos et al., 2018). It is also possible that parasite dormancy may play a role in drug resistance (Sánchez-Valdéz et al., 2018).

### Host Factors in Parasite Susceptibility and Resistance

As in many diseases, the outcome of *T. cruzi* infection is determined not only by the pathogen, but also by the host. We tend to focus on immunity but there are other intrinsic (innate) factors that may also contribute to susceptibility and resistance. These known and unknown attributes are bundled in the vague term “genetic factors.” Beyond the genetic factors there is also host nutritional status, possible presence of coinfections, and other environmental factors that may influence outcome. This concept is best exemplified by the finding that most *T. cruzi*-infected individuals have no clinical signs or symptoms of infection—ever. Regarding host genetic background, T lymphocytes in chronic patients with no clinical disease have a high frequency of CD4^+^ and CD8^+^ T cells expressing HLA-DR and CD45RO (Dutra et al., 1994), with little to no costimulatory CD28 (Dutra et al., 1996; Menezes et al., 2004; Albareda et al., 2006). This profile positively correlates with the expression of the regulatory cytokine IL-10 (Menezes et al., 2004) and also with the presence of CTLA-4, a costimulatory molecule which leads to T cell modulation (Souza et al., 2007). Since CD8^+^ T cell destruction of parasitized cells can lead to tissue inflammation and clinical disease, it is possible that immunoregulatory mechanisms in these patients prevent pathology and facilitate lifelong indeterminate, subclinical disease. The balance between proinflammatory and anti-inflammatory immune responses is central to the outcome of infection. Although a pro-inflammatory adaptive immune response is necessary to control *T. cruzi*, immunoregulation is necessary later on to prevent tissue destruction and possible subsequent autoimmune damage (Bonney and Engman, 2015). In this way, IL-10 plays an essential modulating role in controlling disease development. The ability to express IL-10 at sufficiently high levels may be genetically determined and may influence disease outcome. Studies in experimental models of *T. cruzi* infection demonstrate the influence of host immune response in the outcome of infection. BALB/c mice, which develop a Th2-skewed response upon *T. cruzi* infection, are hypersensitive to infection and do not survive the acute phase. In contrast, C57BL/6 mice, which develop Th1 immunity through the IL-12/IFN-γ/iNOS axis, control the parasite and show low parasitemia and mortality during the acute phase (Michailowsky et al., 2001).

## Toward Precision Health Management of Chagas Disease

As described above, *T. cruzi* infection and Chagas disease are highly complex. At the present time, no single factor or combination of factors can predict disease outcome or response to therapy in an infected individual. The assignment of *T. cruzi* strains to DTUs and the assessment of a person's HLA haplotype and other immunogenetics are starting points. In chronic infection it is often not possible to isolate parasites for analysis and, even if successful, the parasite isolated may not represent all parasite clones present in the patient, which might have different pathogenic potential. Detection of parasites in chronically infected individuals is most frequently accomplished by PCR (Schijman et al., 2011) and this method has also been used to monitor the effect of therapy on parasite persistence (Morillo et al., 2015; Sulleiro et al., 2019). Unfortunately, the sensitivity of PCR is problematic—only 60% in the large BENEFIT Trial of several thousand patients (Morillo et al., 2015). Although there are many possible reasons for this, a likely reason is suboptimal sampling. Perhaps a proteomic approach would be better and recent work detecting *T. cruzi* antigens in circulating immune complexes from infected individuals is promising (Ohyama et al., 2016). A major breakthrough in patient management would be the ability to assess the distribution of parasites in the body, much as is done today with nuclear medicine scans for cancer. We can do this in mice employing bioluminescent imaging of engineered luminescent parasites (Hyland et al., 2008) but obviously this is not possible in patients. Interestingly the infection in mice is highly dynamic with migration of parasite foci around the body over time (Lewis et al., 2014). Development of approaches to image parasites in an infected person to determine location(s) and burden would enhance the care of Chagas patients if these are found to be linked to organ-specific dysfunction. Another would be the detection of parasite-free *T. cruzi* DNA in blood or body fluids, much as is done today for circulating tumor cell DNA. In this way it is theoretically possible to genotype all parasite clones in an infected individual, perhaps quantitatively, by next generation sequencing (NGS), in the absence of circulating parasites. This is an active area of investigation in a number of laboratories (Domagalska and Dujardin, 2020).

A more refined genetic analysis of *T. cruzi* than the DTU system will no doubt emerge through large scale NGS whole exome or genome analysis of parasites and integration of this information with detailed clinical information and patient outcomes, including response to treatment. In this way, the complex interplay between parasite and host genetics that ultimately determines the outcome of infection might emerge. This may not happen tomorrow, but it is a major goal of the medical field as applied to many diseases, both infectious and otherwise. Unfortunately genome wide association studies have not been successful in identifying gene polymorphisms associated with disease progression (Deng et al., 2013). Regarding drug treatment, a comprehensive, systems approach to parasite and host will essentially allow a pharmacogenomic approach to treatment, much as is done today for personalized treatment decisions for cancer, arrythmias and pain management (Wang et al., 2011).

How will this happen? Until now, associations between biological data and biological behaviors were deduced from simultaneous consideration of small numbers of data features from a laboratory experiment or clinical trial. This limitation has hindered our understanding of polygenic diseases like diabetes and coronary heart disease. The advent of machine learning now allows the simultaneous analysis of hundreds or even thousands of features across a very large number of biological samples employing supercomputing to identify relationships among the features (Rajkomar et al., 2019). For some applications, the machine needs to be “trained,” for example by “learning” the associations of histologic images with cancer types. If all goes well with the training, the computer can then type the cancer with high accuracy (Esteva et al., 2017; Gertych et al., 2019). Extending the histology example further, there is information in a complex image like tissue histology that reflects underlying genetic modifications, such as DNA methylation, and machine learning can identify those subtleties in a way that the human eye never could (Zheng et al., 2020). This approach can also be applied to molecular data *de novo* for gene discovery (Wood et al., 2018). Ultimately, the promise of precision health will be realized by the application machine learning to a wider variety of data features and, for Chagas disease, this means clinical data, basic patient information, including demographics, baseline genome sequence, behavioral and physiological data and, of course, genomic information of the parasite clone(s) infecting the person. We believe that it is only a matter of time when this will be science and not just science fiction.

**Figure 1:**
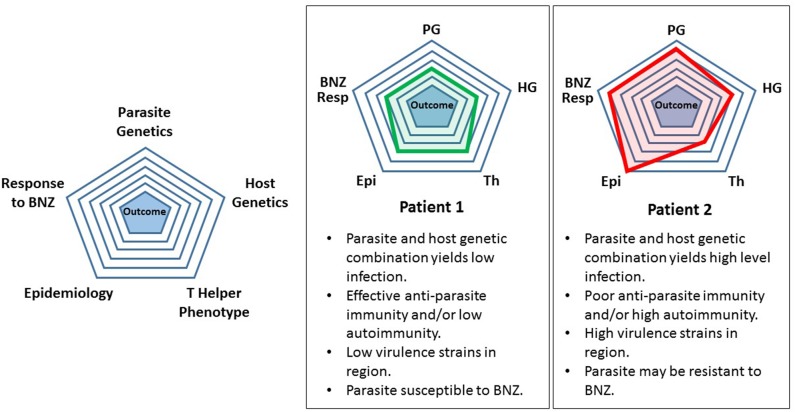
Preliminary simplified model for assessing a *Trypanosoma cruzi*-infected individual. See text for description and discussion.

As an intermediate step between the present and future, we propose a highly simplistic theoretical approach to categorizing *T. cruzi* infection and treatment. Refinement of this model over time by adding levels of sophistication might eventually yield a useful tool for patient management. For example, considering five attributes derived from host or parasite it is possible to generate a pictorial representation of the infection (Figure 1). This is based loosely on the modeling of Santi-Rocca (Santi-Rocca et al., 2017). Each attribute—parasite genetics, host genetics, T helper phenotype, epidemiology, and response to BNZ can be “scored” from 1 to 5, with 1 corresponding to the lowest level of a particular attribute and 5 the highest. When applied to two different infections, Patient 1 having a low-level, chronic infection with no clinical disease and Patient 2 having significant cardiomyopathy, a pictorial representation of the infection can be generated. The attributes are listed below each patient in clockwise order from the top. Clearly this is so simplistic that it is not useful today. These attributes are not really “scorable” in this way and do not correlate in the manner shown in these examples. However, simplistic, this approach does give a preliminary glimpse into a future analytic scheme that shows how specific features of host and parasite might contribute the ultimate outcome of infection and responsiveness to treatment.

In terms of refinement, the field really does need to move beyond studies of laboratory strains to analyze strains present in patients and insects in a systematic and non-biased way through NGS. It is possible that the DTU structure will have value over time since if nothing else DTU by definition reflects the genetic relatedness of strains. We foresee a day when genotyping of the strain(s) present in each patient will become part of the standard workup of *T. cruzi* infection and that NGS genotyping of circulating parasite DNA will solve this problem. The premise underlying this entire discussion is that, ultimately, disease outcome and response to therapy can be predicted based on the genotypes of parasite and host, both as independent factors and in combination. The latter notion is based on the well-known fact that an individual parasite strain shows differential virulence depending on the host, and that an individual host has different disease outcome depending on the parasite strain. This complexity is challenging but no more so than in many other polygenetic diseases affecting millions.

## Conclusion

Since its discovery more than 100 years ago (Chagas, 1909), Chagas disease has proven to be a major clinical and public health challenge due to the extreme heterogeneity in the outcome of infection, the wide range of mammalian hosts and reservoirs, the large geographic range of its triatomine insect vectors, worldwide migration of infected individuals, and paucity of drugs. While we have discussed the potential future for disease diagnosis, prognosis and patient management, the ultimate solution is the development of an effective and curative treatment having low toxicity. Better yet, a safe and effective vaccine that provides sterilizing immunity or even immunity sufficient to minimize the parasite burden to prevent clinical disease. Science and medicine are developing rapidly and we are hopeful that someday *T. cruzi* and Chagas disease will be considered manageable infections, much like the viral and bacterial infections that were previously deadly and now are managed through vaccination and effective drug treatments.

## Author Contributions

All authors listed have made a substantial, direct and intellectual contribution to the work, and approved it for publication.

## Conflict of Interest

The authors declare that the research was conducted in the absence of any commercial or financial relationships that could be construed as a potential conflict of interest.
